# *Rickettsia sibirica* Isolation from a Patient and Detection in Ticks, Portugal

**DOI:** 10.3201/eid1207.051494

**Published:** 2006-07

**Authors:** Rita de Sousa, Conceição Barata, Liliana Vitorino, Margarida Santos-Silva, Carlos Carrapato, Jorge Torgal, David Walker, Fátima Bacellar

**Affiliations:** * Instituto Nacional de Saúde Dr Ricardo Jorge, Águas de Moura, Portugal;; † Hospital do Espírito Santo-Évora, Évora, Portugal;; ‡ Faculdade de Ciências da Universidade de Lisboa, Lisbon, Portugal;; § Instituto da Conservação da Natureza, Mértola, Portugal;; ¶ Universidade Nova de Lisboa, Lisbon, Portugal;; # University of Texas Medical Branch, Galveston, Texas, USA

**Keywords:** *Rickettsia*, spotted fever group, Rickettsia sibirica (strain mongolotimonae), Rhipicephalus pusillus, Rhipicephalus bursa, ticks, Portugal, lymphangitis, research

## Abstract

We report the first isolation of *Rickettsia sibirica* (strain mongolotimonae) from the blood of a patient and detection by polymerase chain reaction (PCR) of the rickettsia in a *Rhipicephalus pusillus* tick collected from a dead mongoose (*Herpestes ichneumon*) in the Alentejo region, Portugal. We describe also the first PCR detection of a new *Rickettsia* strain that is related to *R. sibirica*.

## Abstract 2

First *R. sibirica*–related strain is detected.

*Rickettsia sibirica* (strain mongolotimonae), initially named strain HA-91, was originally isolated from a *Hyalomma asiaticum* tick collected in the Alashian region of Inner Mongolia in 1991 (*1*). Since then, this emerging strain has been detected in other *Hyalomma* species, such as *H. truncatum* and *H. excavatum*, and in different areas of the world (*2**,**3*). In 1996, the first human case of infection caused by this rickettsia was described in France (*4*). This new strain was isolated from the blood and the skin of a patient admitted in March to the Hospital La Timone in Marseille. The patient had a mild illness with an eschar, rash, and fever. The unusual aspect of the case was its occurrence in March, when Mediterranean spotted fever (MSF) is rarely reported. Subsequently, other human cases were described in France, and diagnosis was confirmed by rickettsial isolation or polymerase chain reaction (PCR) detection of the agent in eschar and serum specimens. Cases outside of France have been reported in South Africa and Greece (*3**–**5*).

In Portugal, the only previously recognized rickettsioses were caused by strains of *R. conorii* complex and *R. typhi* (*6**,**7*). However, *R. slovaca*, *R. aeschlimannii*, and *R. helvetica* have been isolated and detected by PCR in Portuguese ticks (*8*). We report the first isolation of *R. sibirica* (mongolotimonae strain) in Portugal from the blood of a patient with an initial clinical diagnosis of MSF and the detection of this rickettsia by PCR in a tick from the same region.

## Case Report

A 73-year-old woman was admitted to Espirito do Santo Hospital in Évora, Alentejo region, on August 18, 2004. No history of travel, tick exposure, or direct contact with domestic animals was reported.

Before admission, the patient sought treatment from her family physician with redness and swelling of the third right toe. She was treated with 5 mg amlodipine. Three days later, her clinical symptoms had progressed. She exhibited fever, myalgia, prostration, and anorexia and was admitted to the hospital. On physical examination, the patient had a nonpuritic, generalized, erythematous, maculopapular rash involving the entire body, including the palms and soles. She was alert and oriented. Her mucous membranes appeared normal, and she had no jaundice or cyanosis. Physical examination found no difficulty in breathing, and her vital signs included temperature 39.6°C, respiratory rate 24 breaths/min, heart rate 81 beats/min, and blood pressure 156/72 mm Hg. Her heart and lungs were normal on examination. Her abdomen had normal peristaltic sounds, and she had no pain on superficial or deep palpation. The patient had a small, deep purple lesion on the anterior aspect of her right third toe. A presumptive diagnosis of MSF was made, and treatment was initiated with penicillin G and 110 mg doxycycline, twice a day for 7 days; 48 hours later the patient was afebrile, and the rash had disappeared.

Laboratory evaluation showed a leukocyte count 7.8 × 10^3^/μL with 86.4% neutrophils, hematocrit 42%, platelet count 177,000/μL, serum creatinine 1.0 mg/dL, alanine aminotransferase 93 IU/L, aspartate aminotransferase 116 IU/L, total bilirubin 0.8 mg/dL, creatine phosphokinase 267 IU/dL, lactate dehydrogenase 1,057 IU/L, and C-reactive protein 18.23 mg/dL. The chest radiograph did not show consolidation or other abnormality. Although the patient's condition gradually improved, her hepatic enzymes remained elevated.

## Materials and Methods

### Human Study

#### Isolation of Rickettsiae

A blood sample (5 mL) was collected from the patient in a sterile heparinized vacutainer (6 days after the onset of illness). The blood was left to sediment for 1 h, and the plasma, buffy coat, and erythrocytes were separated and stored in 1.8-mL tubes (Nunc) at -80°C. The buffy coat was added to a single shell vial seeded with Vero cells (African green monkey fibroblast cells) and centrifuged at 700 × g for 1 h in Eagle's minimal essential medium (MEM) at 22°C by using the centrifugation-enhanced shell-vial technique (*9*). After centrifugation, the supernatant was discarded, and 1 mL MEM was added. The shell vial was incubated at 32°C, and on day 6, the cell monolayer from the shell vial was scraped with glass beads and was transferred to a confluent monolayer of Vero cells in a 25-cm^2^ culture flask, but no Gimenez staining or immunofluorescence assay (IFA) was conducted. For a period of 6 days, the monolayer was scraped daily, and a slide was prepared for Gimenez staining as previously described (10At day 5, when microscopy showed rickettsial growth by Gimenez staining, a new slide was prepared to identify the bacterial growth by IFA, by using polyclonal human sera (pool of positive sera from patients containing immunoglobulin G (IgG) antibodies against *R. conorii*) as previously described (*11*). The cells were scraped with glass beads, 3 aliquots were stored in 1.8-mL tubes (Nunc) at -80°C, and the fourth was used to propagate the rickettsial isolate into a fresh confluent monolayer of Vero cells in a 25-cm^2^ culture flask. After 8 days, the cells of the flask were scraped, and the cell suspension was harvested, centrifuged at 5,000 rpm for 30 min, and resuspended in phosphate-buffered saline (PBS) for DNA extraction.

Serologic testing of the patient's acute-phase serum (i.e., collected 6 days after the onset of illness) was performed by indirect IFA with antigens *R. conorii* Malish strain and *R. typhi* prepared at the Instituto Nacional de Saúde Dr Ricardo Jorge as previously reported (*11*). IgM titers >64 and IgG titers >128 for *R. conorii* and *R. typhi* were considered diagnostic of spotted fever or typhus rickettsiosis, respectively. After the isolate was characterized, the patient's serum was tested again by using the new *R. sibirica* (mongalotimonae strain) isolate as antigen.

#### DNA Extraction, PCR, and Sequencing

DNA was extracted from 200 μL of PBS cell suspension by using the DNeasy tissue Kit (Qiagen, Hilden, Germany) according to the manufacturer's recommendations. PCR assays targeting the rickettsial genes for citrate synthase (*gltA*) and outer membrane protein A (*ompA*) were performed with specific primers. For citrate synthase gene (*gltA*), novel primers were designed, RpCS.415 (forward, 5´ GCTATTATGCTTGCGGCTGT 3´) and RpCS.1220 (reverse, 5´ TGCATTTCTTTCCATTGTGC 3´), which amplify a 806-bp fragment. For the *ompA* gene, the primers Rr190.70p and Rr 190.602n, which amplify a 532-bp fragment, were used as previously described by Regnery et al. (*12*). Samples that yielded PCR products were confirmed by using a PCR assay incorporating the 120-M59´ and 120–807´ primer pair, which amplifies a 833-bp fragment of the *ompB* gene of *Rickettsia*, as previously described by Roux and Raoult (*13*). PCR was performed in a 50-μL reaction mixture containing 25 μL of the High Fidelity PCR Master Kit buffer (Roche Diagnostics, GmbH, Mannheim, Germany), 2 μL of each primer at 0.2 μmol/L, and 5 μL genomic DNA. Amplification was performed in a DNA thermocycler (T-3 thermoblock T, Biometra, Goettingen, Germany) under the following conditions: 2 min of initial denaturation at 94°C, then 35 cycles of 94°C for 30 s, 58°C (*gltA*) or 52°C (*ompA*, *ompB*) for 30 s, and 72°C for 90 s. Amplification was completed by holding the reaction mixture at 72°C for 7 min to allow complete extension of PCR products. For each reaction, a negative control (water) was included, and no positive control was used to avoid contamination. Five microliters of the PCR products were resolved by electrophoresis in 1.2% agarose gel, stained with ethidium bromide, and examined by UV transillumination. PCR products were purified by using the QIAquick Spin PCR purification kit (Qiagen) as described by the manufacturer. The purified PCR products were sequenced in an ABI automated sequencer (Applied Biosystems, Foster City, CA, USA) by using the ABI PRISM Big Dye Terminator Cycle Sequencing Ready Reaction Kit (Applied Biosystems), according to the protocols supplied by manufacturers. All sequences were determined by the consensus of the forward and reverse sequence analysis. The sequences of the *gltA*, *ompA*, and *ompB* amplicons were aligned with the corresponding sequences of other *Rickettsia* species available in GenBank/EMBL database, by using BLASTN software (*14*).

#### Phylogenetic Analysis

Phylogenetic relationships were inferred by using PAUP version 4b10 (*15*). For *ompA* gene analysis, a phylodendrogram was constructed by the neighbor-joining method, and distance matrixes were calculated by using the Kimura 2-parameter model to correct for multiple substitutions (*16**,**17*). Bootstrap values for the trees were obtained from 1,000 randomly generated trees.

### Tick Study

#### Collection of Ticks

A total of 175 ticks were collected in different locations in the Alentejo region, for example, Beja, Ourique, Mourão (Alqueva Dam), and Mértola (Natural Park of Guadiana), during 2004. The species were identified on the basis of morphometric characteristics by 1 author (M. Santos-Silva), and ticks were kept in individual sterile tubes without any additive at –80°C until further processed.

#### DNA Extraction, PCR, and Sequencing

Ticks were washed for 5 min in iodinated alcohol and then in sterile distilled water for 5 min before being dried on sterile filter paper. DNA was extracted from ticks by using alkaline hydrolysis, as described previously (*18*). DNA from each tick was used as template in PCR assays targeting the rickettsial gene for citrate synthase (*glt*A) by using the same primer set (RpCS.415 and RpCS.1220) that was used for characterization of the human isolate. Samples that yielded PCR products were subsequently confirmed by another PCR by using primers Rr190.70p and Rr 190.602n for *ompA*. In 2 of the PCR-positive ticks, the presence of rickettsia was confirmed by using the primers 120-M59´and 120–807 for *ompB* to generate additional sequence data (*14*). PCR amplification, sequencing, and data analysis were performed as the protocol described above for the characterization of the rickettsial isolate obtained from the patient.

#### Nucleotide Sequence Accession Numbers

The GenBank nucleotide sequence accession numbers for partial sequences of *gltA*, *ompA*, and *ompB* genes generated in this study as follows. For PoHu10991, they are DQ423368, DQ423365, and DQ423364, respectively; for PoTiRb169, they are DQ423369, DQ423366, and DQ423363, respectively; and for PoTiRp53, they are DQ423370, DQ423367, and DQ423362, respectively.

## Results

An isolate was obtained from the blood of the patient. The rickettsia was first detected in culture by Gimenez staining and IFA, and then the established isolate was characterized by PCR assays and sequencing. By BLAST analysis, the *gltA* sequence of the human isolate (PoHu10991) was 99.8% (653/654 bp), similar to that of *R. sibirica* (U59731). The *ompA* sequence was 99.8% (480/481 bp), similar to that of *Rickettsia* sp. HA-91 strain (U43796), and the *ompB* sequence was 100% (776/776), similar to that of *R. sibricia* (mongolotimonae strain) (AF123715). These data show that our isolate is definitively *R. sibirica* mongolotimonae strain.

The patient's acute-phase serum contained neither IgM nor IgG antibodies that reacted with *R. conorii* or *R. typhi* antigen by IFA. A second serum sample was not available.

**Figure 1:**
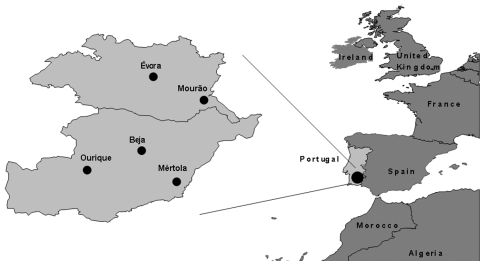
Site of tick collection in the Alentejo region.

Of the 175 ticks collected in nonsystematic schedule from March through August in different locations in the Alentejo region (Figure 1), 5 were *Rhipicephalus bursa*, 12 *R. turanicus*, 20 *R. pusillus*, 68 *R. sanguineus*, 59 *Hyalomma lusitanicum*, and 11 *Dermacentor marginatus*. The ticks were collected from different animals including Egyptian mongoose (*Herpestes ichneumon*), sheep (*Ovis aries*), cow (*Bos tauros*), dog (*Canis familiaris*), and vegetation (Table 1 and Table 2). Rickettsial DNA was detected in 12 (6.9%) of the 175 ticks examined. Nine *Rhipicephalus* spp. and 3 *D. marginatus* contained rickettsiae detected by PCR (Table 2). All 59 *H. lusitanicum* were negative for rickettsial DNA. DNA from 1 male tick, identified as *R. pusillus*, collected in March from a dead Egyptian mongoose (*Herpestes ichneumon*) in the Alqueva Dam region (Figure 1), contained a rickettsia exhibiting nucleotide sequence of *gltA* 99.8% (654/655 bp) similar to *Rickettsia* sp. HA-91 (U59731). For *ompA* the sequence was 100% (484/484 bp) similar to *Rickettsia* sp. HA-91 (U43796), and the *ompB* sequence was 100% (660/660 bp) similar to *R. sibirica* mongolotimonae strain (AF123715). This Portuguese strain was designated PoTiRp53.

**Table 1:** Number of *Rhipicephalus* spp. collected in the Alentejo region and identified rickettsiae

Month/ site	Origin	Tick species (no., sex)*	Identified rickettsia
March			
Beja	Egyptian mongoose	*Rhipicephalus turanicus* (2M)	1 *Rickettsia* bar29
Mourão	Vegetation	*R. turanicus* (1M)	1 *Rickettsia* bar29
	Egyptian mongoose	*R. pusillus* (13F;7M)	1 *R. sibirica*
May			
Mértola	Dog	*R. sanguineus* (8F; 22M)	2 *Rickettsia* bar29
	Vegetation	*R. sanguineus* (2F; 4M)	–
		*R. turanicus* (2F)	–
	Cow	*R. sanguineus* (3F)	–
		*R. bursa* (2M)	–
	Sheep	*R. sanguineus* (1F)	–
		*R. bursa* (1F; 2M)	1 *Rickettsia* sp.
		*R. turanicus* (2F; 4M)	1 *Rickettsia* bar29
Ourique	Dog	*R. turanicus* (1M)	1 *Rickettsia* bar29
Beja	Dog	*R. sanguineus* (6 M)	1 *Rickettsia* bar29
		*R. sanguineus* (1M)	–
June			–
Ourique	Dog	*R. sanguineus* (10F; 7M)	–
August			
Mértola	Dog	*R. sanguineus* (2F, 2M)	–

*F, female; M, male.

**Table 2:** Number of *Hyalomma lusitanicum and Dermacentor marginatus* collected in the Alentejo region and identified rickettsiae

Month/ site	Origin	Tick species (no., sex)*	Identified rickettsia
March			
Mourão	Vegetation	*H. lusitanicum* (10F; 6M)	–
April			
Mértola	Vegetation	*D. marginatus* (2F; 3M)	–
May			
Mértola	Vegetation	*H. lusitanicum* (23F; 15M)	–
	Cow	*H. lusitanicum* (1F; 3M)	–
	Sheep	*H. lusitanicum* (2M)	–
	Vegetation	*D. marginatus* (6F)	*Rickettsia* sp. RpA4

*F, female; M, male.

A second rickettsia species designated PoTiRb169 was identified in 1 *R. bursa* tick. The *gltA* sequence was 99.2% (655/660) similar to that of *R. sibirica* (U59734). The *ompA* was 97.5% (504/517) similar to that of *Rickettsia africae* (U83436), and the *ompB* was 98.6% (789/800) similar to that of *R. africae* (AF123706).

*R. massiliae* (bar 29 strain) was detected in 4 *R. turanicus* and 3 *R. sanguineus* ticks. *Rickettsia* sp. strain RpA4 was detected in 3 *D. marginatus* ticks. These data will be presented in a separate report.

**Figure 2:**
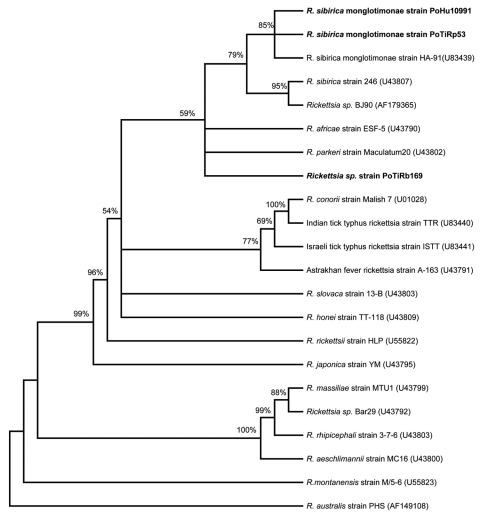
Unrooted consensus tree inferred from 1,000 replicated trees based on partial *ompA* gene sequence. Evolutionary distances were estimated by the Kimura 2-parameter model, and phylogenetic relationships were assessed by neighbor-joining method. Bootstrap values are indicated at the nodes. Branches with bootstrap values <50% are collapsed. Portuguese *Rickettsia* strains are indicated by **boldface** type. GenBank accession numbers are indicated for each rickettsia.

Phylogenetic analysis based on the *ompA-*encoding gene showed that the human isolate PoHU10991 is most closely related to *R. sibirica* monglotimonae strain (GenBank accession no. U83439) as well as to the strain PoTiRp53, which was detected in *R. pusillus*. This cluster is supported by a high bootstrap value (>85%) (Figure 2). *Rickettsia* sp. PoTiRb169 strain is related to the *R. sibirica* cluster; however, the bootstrap value is low (52%), which means that this genotype was not accurately identified.

## Discussion

To our knowledge, this is the first reported isolation of *R. sibirica* (strain mongolotimonae) from a patient's blood in Portugal. The patient, whose condition was originally diagnosed as MSF, sought treatment with 1 lesion on her toe that resembled a tick bite; fever; and maculopapular rash; these signs occurred in the month with the highest incidence of MSF. Therefore, no one suspected, on epidemiologic and clinical grounds, that she had a rickettsiosis that was different from MSF. The blood specimens were sent to our laboratory for routine serodiagnosis and blood culture. Our laboratory had long experience in isolation of rickettsiae from the blood of patients (>80 strains isolated) and performed the usual procedure for blood samples (*19*). The blood isolation was a marked achievement in identifying *R. sibirica* (strain mongolotimonae) because even if the patient had antibodies but no rickettsial isolation, we would have problems differentiating the illness from other rickettsial infections, since the serum cross-reacted with *R. conorii* antigen and in our laboratory, IFA for this rickettsia was not available. Determining how many days were necessary for the patient's seroconversion would be useful, but a second serum sample was not available.

This patient exhibited clinical signs and symptoms similar to MSF, and she did not manifest lymphangitis or enlarged lymph nodes, a clinical feature that has been proposed as typical of *R. sibirica* (strain mongolotimonae) infection because it occurred in 44% of French patients with this infection (*20*). Characterizing and differentiating rickettsioses only on the basis of clinical manifestations is difficult since the same agent can exhibit different signs, depending on the host. In fact, in Israeli spotted fever, a study of Portuguese patients found no significant differences in the presence of eschars among patients infected with different strains of *R. conorii*. In contrast, in most cases reported from Israel, the eschar is rare or absent (*19*). Furthermore, infections caused by different *Rickettsia* spp. can cause the same sign, and not only the typical signs; for example, lymphangitis has been reported in African tick bite fever, *R. heilongjiangensis* infections, and as a reaction to argasid tick bites (*21**–**23*). Among 12 patients with *R. sibirica* mongolotimonae strain infection, 3 (25%), from Algeria, South Africa, and Portugal, were bitten by a tick on the foot, and in the last 2 patients, the eschar was found between the toes. Series of MSF cases have reported eschars on the leg but not in the foot (*6**,**19*).

Most of the cases caused by *R. sibirica* (strain mongolotimonae) reported in France have occurred in the spring, including only 1 case in early July, whereas the patients from South Africa and Greece were ill in winter. In contrast, our case occurred in August during the peak of the MSF season. Probably the occurrence of these cases in different months could be related to the differences in seasonal activity and population dynamics of different vectors. In countries such as Mongolia, Greece, Niger, and South Africa, *R. sibirica* is likely transmitted by *Hyalomma* ticks, but we report for the first time that a new tick host, *Rhipicephalus pusillus*, might be also implicated in the transmission of this rickettsia in Portugal. This finding alerts us to the possibility that the number of tick genera and species infected with *R. sibirica* (strain mongolotimonae) may be larger than the originally described *Hyalomma* spp. This fact is not surprising since *R. sibirica*, the agent of North Asian tick typhus, has been found in numerous different genera and species, including *Hyalomma* spp., *Dermacentor* spp., and *Haemaphysalis concinna* (*20*).

*R. pusillus* is present in all districts in the south of Portugal throughout the year, with a higher density from March to October (*24*). Also during this period, *R. sanguineus*, the vector implicated in transmission of the strains of *R. conorii*, exhibits higher density and activity. Although *R. sibirica* has been detected in *R. pusillus* in March, this species is also highly prevalent in August, when the human case was described. The higher density of other *Rhipicephalus* spp. such as *R. turanicus*, occurs in April and May, and for *R. bursa*, from May to August. In general, in Portugal, *Rhipicephalus* spp. are more prevalent in spring and summer. *Hyalomma* spp. are found in all seasons but are more prevalent from the end of summer through autumn and winter. *D. marginatus* prefers the cooler months (*24*). That *H. lusitanicum* ticks were not determined to be infected does not mean that they might not also be vectors of rickettsiae. This species has previously been found to harbor rickettsialike organisms, and *H. marginatum* has been found to be infected with *R. aeschlimannii* (*8*). All these ticks have been detected on humans in Portugal (*25*).

The role of *Rhipicephalus* spp. in the transmission of different rickettsiae in Portugal is corroborated by the finding of a new rickettsial strain, named PoTiRb169, detected in *R. bursa*. Although this strain differs from *R. sibirica* (strain mongolotimonae), it is closely related to this group.

The *ompA* phylogenetic analysis confirmed that rickettsial strain PoHu10991, isolated from a Portuguese patient, and PoTiRp53 strain, isolated from *R. pusillus*, are similar to *R. sibirica* mongolotimonae strain HA-91. An identical cluster is obtained when a phylogentic tree is inferred from *gltA* gene sequences (data not shown). The *ompA* phylogeny has a low bootstrap value for the branching of *Rickettsia* sp. PoTiRb169. To establish the correct identification of this rickettsial strain according to genetic guidelines published by Fournier et al., other gene sequences (*rrs* and *sca4* genes) must be obtained, and more phylogenetic analysis must be performed (*26*).
